# Preparation, characterization, stability, and application in curcumin of the emulsion gels stabilized with coconut cake albumin modified by ultrasonication and carboxymethylation

**DOI:** 10.3389/fnut.2026.1943175

**Published:** 2026-08-26

**Authors:** Yong Yang, Meiling Gong, Jiapeng Duan

**Affiliations:** 1 Shanxi Province Cancer Hospital, Taiyuan, China; 2 Shanxi Hospital Affiliated to Cancer Hospital, Chinese Academy of Medical Sciences, Taiyuan, China; 3 Cancer Hospital Affiliated to Shanxi Medical University, Taiyuan, China; 4 Food Science College of Shanxi Normal University, Taiyuan, China

**Keywords:** carboxymethylation, characterization, coconut cake albumin, curcumin bioaccessibility, emulsion gel, stability, ultrasonication

## Abstract

**Background:**

Curcumin has applications in food and medical industries, but its stability and bioaccessibility are poor. Coconut cake albumin (CCA) has potential as a component of the curcumin-loading system; however, relative data are scare.

**Methods:**

Herein, emulsion gels were formed using coconut cake albumin modified by ultrasonication and carboxymethylation (CCA-UC) in this study.

**Results:**

The results evidenced that ultrasonication and carboxymethylation increased the emulsifying capacity (from 67.51 to 143.65 m^2^/g) and emulsion stability (from 70.37 to 87.18%) of CCA by improving its solubility and interface sorption capacity, enhancing the zeta potential, and reducing droplets’ size (*p* < 0.05). CCA and CCA-UC formed compact emulsion gel when concentration was more than 10 g/100 g. Compared with the CCA-emulsion gel, CCA-UC-emulsion gel had more compact and denser gel structure, higher crystallinity (38.65%), bound water content and viscosity, and lower energy storage (*G’*), loss modulus (*G”*), and loss factor. Furthermore, CCA-UC-emulsion gel showed superior thermal and oxidative stability, and higher chewiness (35.83 g), springiness (0.90), hardness (67.44 g), and cohesiveness (0.85) than CCA-emulsion gel. Additionally, CCA-UC-emulsion gel exhibited superior intestinal digestion rate, higher curcumin encapsulation (89.64%) and loading efficiency (204.01 μg/g), better photostability, and superior bioaccessibility (49.08%). However, more specific mechanisms should be investigated in the further work.

**Conclusion:**

These findings revealed that ultrasonication assisted with carboxymethylation was an effective way to improve the emulsion gel properties of CCA and increase the bioaccessibility of curcumin. However, more specific mechanisms should be investigated in the further work.

## 1 Introduction

Emulsion is widely used in food industry but it is mostly thermodynamically unstable, which limits their widespread applications (1). By contrast, emulsion gels have adjustable elasticity and better stability relative to emulsions and gels (2). Emulsion gels are semi-solid or solid materials with both the rheological properties of emulsions and the mechanical properties of gels (3). Prior studies demonstrated that emulsion gels significantly enhanced the stability and bioavailability of lutein esters, curcumin, lycopene, and other bioactive compounds (1, 4). In recent decades, the effects of curcumin in anti-inflammatory, improving cardiovascular and cerebrovascular conditions, and anti-cancer have been extensively proven by numerous clinical trials (4). However, the usage of curcumin in food industry is limited, because the bioavailability and aqueous solubility of curcumin are low, and its stability against light, thermal treatment, and alkali and acidic conditions is poor (5). Pickering emulsion is an effective way to improve the bioaccessibility of curcumin (6, 7), but this system is thermodynamically unstable. Although some emulsion gels based on proteins (such as egg white protein, soybean protein, and zein), or protein-polysaccharide and protein-polyphenols complexes have been used to delivery curcumin for improving its stability and bioavailability (4, 8, 9), data about curcumin delivery system based on modified proteins is limited.

More importantly, emulsifiers and gelatinizers are both crucial for the formation of a stable emulsion gel. Compared to the chemically synthesized emulsifiers and gelatinizers, food-derived proteins and their derivatives are better candidates for their better biodegradability and biocompatibility, and superior safety (10). However, most natural proteins exhibit relatively poor emulsion properties than chemically synthesized emulsifiers such as Span 80, Tween 80, and sodium caseinate (11). To improve the emulsion gel properties of proteins, glycosylation, polyphenol conjugation, carboxymethylation, ultrasonication, and high-speed shearing have been employed (12–14). However, data about the effect of these modification methods, especially the combination of these methods on the emulsion gel properties of the proteins are little.

Coconut (*Cocos nucifera* L.) cake is a by-product of coconut oil and is almost used for feed processing (15). The protein content of coconut cake is 12–21 g/100 g, of which the content of albumin is approximately 35 g/100 g (16). The emulsifying property of coconut cake albumin (CCA) was superior than soy globulin (17). Tian et al. (18) found that ultrasound remarkably enhanced the emulsifying properties of coconut protein. The results of our preliminary experiments showed that and ultrasonication and carboxymethylation significantly improved the emulsifying activity (from 84.68 to 124.18 m^2^/g) and emulsion stability (from 60.75 to 87.18%). Compared with coconut globulin, CCA is more hydrophilic, for the hydrophilic amino acids of CCA are predominate, specifically glutamic acid, arginine, and asparagine account for 23.8, 17.65, and 6.95 g/100 g (19). Therefore, carboxymethylation—a chemical modification that enhances molecular polarity—is a rational strategy to improve the emulsifying capacity of CCA. However, the effects of these modifications on the emulsion gel properties of CCA were not investigated. Herein, CCA modified by ultrasound and carboxymethylation was used to prepare an emulsion gel delivery system to improve the stability, digestion rate, and bioaccessibility of curcumin. The structural, textural, rheological, and digestive properties, and stability of the emulsion gel were studied. Moreover, the underlying mechanism how ultrasonication and carboxymethylation improved the emulsifying and emulsion gel properties of CCA and the bioaccessibility of curcumin was investigated. This study provides new and more concise strategies to enhance the performance of protein emulsion gels.

## 2 Materials and methods

### 2.1 Materials

Coconut cake was purchased from Gaijing Coconut Trading Co., Zhengzhou, China. Reagents of analytical grade, including petroleum ether II, chloroacetic acid, anhydrous ethanol, sodium hydroxide, disodium hydrogen phosphate, anhydrous calcium chloride, methanol, pepsin (from porcine stomach mucose, 3.0 × 10^3^ U/mg), trypsin (from pig pancreas, 1: 350 U/mg), sodium dodecyl sulfate (SDS), and anhydrous calcium chloride were from Soliabo Chemical Co. Ltd., Tianjin, China. Curcumin (V96324, >95%) was purchased form Huaisu Biological Reagent Ltd. Co., Chengdu, China.

### 2.2 Extraction and ultrasonic treatment of CCA

Coconut cake was defatted using petroleum ether II with a ratio of 1:20 (g/mL) in triplicates, and then crushed and sieved through a 100-mesh sieve (TJ-224, Yushang Sieve Instrument Factory, Shangyu, China) (19). The obtained defatted coconut cake powder (69.5 g) was mixed with 350 mL of deionized water (dH_2_O) and stirred with a rotate speed of 220 r/min using ZDY-V oscillator (Dawu oscillator Co. Ltd., Xuyu, China). After 2 h, the extraction suspension was filtered and the filtrate was centrifuged at 4,350 × *g* for 35 min. The supernatant was dialyzed against dH_2_O using a dialysis membrane with a molecular weight cutoff of 3,500 Da at 4 °C for 48 h. The dH_2_O was changed every 4 h. The retentate was freeze dried using FID75 lyophilizer (Xinyi Dry Instrument Factory, Nantong, China), and CCA was obtained.

Next, 10 g CCA was dispersed in 200 mL of 75% ethanol solution (v/v), and treated using a Ljna-J54 ultrasonic generator (Lingjiang Instrument Factory, Jingzhou, China) at 7.90 W/mL, 40 °C, and 80 kHz (18). The size parameters of the inner tank were 470 × 285 × 285 mm. The treated dispersion was collected and lyophilized using a CNMXS-009 lyophilizer (Hanxin Dryer Instrument, Jinhua, China) to get ultrasonic treated coconut cake albumin (CCA-U).

### 2.3 Carboxymethylation of CCA-U

The carboxymethylation of CCA was done using the same procedures reported by Zheng et al. (20). Briefly, 120 mL of the CCA-U solution (1 mg/mL) was stirred at 120 r/min for 30 min. Concurrently, 5 mL of NaOH (2.23 mol/L) was mixed with 20 mL of 75% ethanol (*v*/*v*) and added to the CCA-U ethanol solution. The reaction system was vortexed at 1,200 r/min for 12 s, followed by heating and alkalization at 53 °C and 205 r/min for 1 h in the ZDY-V oscillator. Subsequently, 1.2 mL of NaOH (2.23 mol/L, dissolved in 85% ethanol) and 1 mL of chloroacetic acid (2.05 mol/L, dissolved in 75% ethanol) were added to the reaction system. The reaction solution was shaken at 205 r/min and 35 °C for 30 min, and then heated at 48 °C and 175 r/min for 150 min. To terminate the reaction, the solution was cooled to 25 ± 1 °C and adjusted to pH 7.0 using 50% acetic acid (*v/v*). The residual chloroacetic acid was removed by centrifugation at 3,000 g for 25 min, and the supernatant was sealed in a dialysis membrane with a molecular weight cutoff of 3,500 Da. After 48 h of dialysis against dH_2_O at 4 °C, the dialysate was lyophilized to obtain coconut cake albumin modified by ultrasonic and carboxymethylation (CCA-UC). The carboxymethyl degree was measured according to the same protocol of Wang et al. (21).

### 2.4 Physicochemical properties

#### 2.4.1 Solubility

CCA dispersions (2 g/100 mL dH_2_O) within pH range of 2–10 was shaken at 25 °C in a HII-7250 triangular flask (Jiangzhe Triangular Instrument factory, Jinhua, China) for 0.5 h (22). After 20 min of centrifugation (3,950 *g*), the protein content in the supernatant was quantified using the Bradford method (23). The percentage of the protein content in the supernatant per 2 g/100 mL was expressed as the solubility (g/100 g).

#### 2.4.2 Emulsifying capacity and emulsion stability

The emulsifying capacity and emulsion stability of CCA and CCA-UC were investigated according to the turbidity method (24) and following the same procedures reported by Wang et al. (22). CCA solutions (1 mg/mL) were adjusted to pH values of 2.5, 4.5, 6.5, 8.5, and 10.5, respectively. Each solution (45 mL) was mixed with 15 mL of soybean oil and homogenized at 17,500 rpm for 2 min using an HTX-II rapid dispersion device (Hangzhou DSIRAD Co. Ltd., Hangzhou, China). After homogenization, 40 μL of the emulsion was drawn out and diluted 500 times using SDS solution (1 g/100 mL). The absorbance at 500 nm of the dilution after 0 and 10 min were record as *A_0_* and *A_t_*. The Equations 1 and 2 were used for quantification of the emulsion stability and emulsifying ability, respectively:

$$\text{Emulsion stability}\;(\%)=A_{t}/A_{0}\times 100\%$$ (1)

$$\text{Emulsifiability}\;(m^{2}/g)=(2.303\times 2\times A_{0}\times f)/(C\times \varphi \times 10,000)$$ (2)

where, 2 and 2.303 are constants; *f*, dilution factor (500); *C*, protein content; *φ*, oil volume fraction (0.25).

#### 2.4.3 Interface sorption ability

The interface sorption ability of CCA and CCA-UC at different pH (2–10) was measured using the centrifugation method from Krstonošić et al. (25). Briefly, the emulsions prepared using CCAs were centrifuged at 7,850 *g* for 20 min. The supernatant was collected. This process was repeated for three times. The supernatant was pooled and used for protein content determination following the Bradford method (23). The difference between the initial and final protein content per the initial protein content was defined as the interface sorption ability.

#### 2.4.4 Particle size and zeta-potential

The volume-area-weighted average size (D_3,2_) and zeta-potential of the emulsions prepared described in section 2.4.3 was measured using a WRINEN1470 laser granularity instrument (Changhe Nano Size Analyzer Factory, Nantong, China) and LJ-159 Micro-Potential meter (Huiwang Potential Instrument Factory, Nanjing, China), respectively.

### 2.5 Preparation of emulsion gels

Using the procedures described by Cui et al. (26), 45 mL of CCA solution with different concentration (final content of 6–12 g/100 g in the gels), 3 mL of sodium alginate (2 g/100 mL), and soy oil (15 mL) were homogenized at 20,000 rpm for 2 min using HTX-II rapid dispersion device (Hangzhou DSIRAD Co. Ltd., Hangzhou, China). Calcium chloride (5.54 mg/mL) was immediately added to form a suspension, and then homogenized at 20,000 rpm for 60 s. The homogeneous liquid was heated at 80 °C for 30 min and placed at 4 °C for 16 h, and then gel was formed.

### 2.6 Structural characteristics

#### 2.6.1 Morphology analysis

Place the emulsion gels based on CCA and CCA-UC (content of 6–12 g/100 g) at room temperature for 3 d, and then photographed using a Konica camera without amplification (27).

#### 2.6.2 Surface microstructure analysis

CCA- and CCA-UC-emulsion gels (2 g) were cut into pieces with a diameter of approximately 1 cm, and then dehydrated in a vacuum dryer. After 48 h, the residual oil droplets were removed by soaking them in ether. The dry gel powder was investigated using a JOLE-JMS-70E50 electron microscope (Electronics Corporation, Osaka, Japan) for apparent morphology and microstructure analysis. Briefly, the sample was pasted on conductive adhesive and then underwent gold spraying (10 nm) (28). The magnification factor was 5,000 with accelerating voltage of 10 kV and scale of 1 μm.

#### 2.6.3 Fourier-transformed infrared spectroscopy

The dry CCA emulsion gel powder (1 mg) prepared as described in section 2.5 and potassium bromide (approximately 100 mg) were thoroughly mixed, ground up, and then compressed into thin slice. The slice was placed at the sample loading platform of a Nicolet CCR-II FT-IR Spectrometer (Thermo Fisher Scientific, Waltham, United States) and analyzed from 4,000 to 400 cm^−1^. The resolution was 4 cm^−1^ (29).

#### 2.6.4 X-ray diffraction

The dry emulsion gel powder (100 mg) prepared in section 2.5 was loaded in the sample tank of ARL EQUINOX 100 *X*-ray diffractometer (Thermo Fisher Scientific, Waltham, United States) (29). The scanning speed, *2θ* scanning range, and current were 10°/min, 5°–70°, and 30 mA, respectively. The lowest intensity (*I_am_*) and maximum intensity (*I_002_*) were determined and Equation 3 was used to quantify the crystallinity:

$$\text{Crystallinity}\;(\%)=(1-\frac{I_{\mathrm{am}}}{I_{002}})\times 100$$ (3)

#### 2.6.5 Intermolecular interactions

Following the method of Han et al. (12), the fresh wet emulsion gel (1 g) was separately dissolved in four kinds of solution, including sodium chloride solution (SA, 0.6 mol/L), 1.5 mol/L of urea solution containing 0.6 mol/L of sodium chloride (SB), 8 mol/L of urea solution containing 0.6 mol/L of sodium chloride (SC), and 8 mol/L of urea solution containing 0.6 mol/L of sodium chloride and 0.5 mol/L of *β*-mercaptoethanol (SD). These four kinds of suspensions were thoroughly stirred at 12,000 rpm for 50 s using the HTX-II rapid dispersion device, and centrifuged at 10,000 × *g* and 4 °C for 0.5 h. The supernatant was used for protein concentration determination using the Coomassie brilliant blue assay (23). The concentration of protein in the supernatant extracted by SA, and the protein concentration differences between SB and SA, SC and SB, SD and SC were defined as the ionic bond, hydrogen bond, hydrophobic interactions, and disulfide bond contents, respectively.

#### 2.6.6 Differential scanning calorimetry

Following the method of Farooq et al. (8), 2 mg of the dry emulsion gel powder was sealed in an aluminum box and loaded in the sample tank of DSC8000 differential scanning calorimeter (PerkinElmer Corporation, Waltham, Massachusetts, United States). The heating rate and the scanning range were 10 °C /min and 25–120 °C, respectively.

### 2.7 Characteristics of the emulsion gels

#### 2.7.1 Water-holding ability

Freshly prepared emulsion gel (approximately 2 g) was precisely weighted (*W_0_*) and transferred into a dry centrifuge tube (10 mL) (4). The centrifuge tub was precisely weighted (*W_1_*) and centrifuged at 4,300 *× g* for 20 min. After being inverted at 25 °C for 0.5 h, a filtrate paper was used to absorb the effluent oil. The total weight of the tub and residual gel was weighted (*W_2_*). The water-holding ability (WHA) was calculated using Equation 4:

$$\mathrm{WHA}\;(g/g)=(W_{1}-W_{2})/W_{0}$$ (4)

#### 2.7.2 Low-field nuclear magnetic resonance

The water distribution within the emulsion gels was analyzed using Low-Field Nuclear Magnetic Resonance equipped with Carr-Purcell-Meiboom-Gill sequence (Brook Technology Co., Ltd., Beijing, China). NMR analysis was done at 32 °C. The RF delay, echo time, and number of scans were 0.5 ms, 0.5 ms, and 8, respectively (26). The relaxation time (T_2_) was calculated consequently.

#### 2.7.3 Texture properties

As per the method of Lin et al. (2), the texture properties of CCA-emulsion gels, including cohesiveness, hardness, chewiness, gumminess, and resilience were determined employing a TA^+^ textural analyzer (Lloyd’s Instruments, AMETEK, Inc., Connecticut, United States) and a P/36 R probe. The operation was done when the pre-, mid-, and operation rates were 2, 1, and 1 mm/s, respectively. The compression rate was 50%, and trigger force was 3 *g*.

#### 2.7.4 Rheological characteristics

The energy storage modulus (*G’*), viscosity, loss factor, and loss modulus (*G”*) of CCA-emulsion gels were investigated on an TA. XTC-20 texture analyzer (Keji Rheometer Instrument Factory, Zhenjiang, China). First, the emulsion gels were placed on the P550P parallel plate and equilibrated at 25 °C (using the T-PPD controller) for 5 min to relax loading stresses (30). The viscosity was determined at shear speed of 0.1–100 s^−1^. During dynamic frequency scanning, the oscillation frequency, linear range, and gap were 0.1–100 Hz, 0.1–100 rad/s, and 1 mm under stress range of 0.1–1,000 Pa, respectively. The rate of temperature changing during heating (25–100 °C) and cooling (100–25 °C) were both 10 °C/min.

### 2.8 Oxidation stability of the emulsion gels

Following the method of Rahman et al. (29), CCAs-emulsion gels were left under 60 °C for 14 d for oxidation stability evaluation. Every 2 days, CCA-emulsion gel (0.1 g) was mixed with 1.5 mL of isooctane and 0.5 mL of isopropanol mix solution. The mixture was vortexed at 1,250 rpm for 15 s, and centrifuged at 3,400 × *g* for 120 s. Then, the upper solution (0.2 mL) was pipped into 2.8 mL of the mixture of butanol and methanol (2,1, v/v). Next, 3.9 mol/L of NH_4_SCN (15 μL) and 0.132 mol/L of FeCl_2_ (15 μL) were added and vortexed at 1,250 rpm for 10 s. The absorbance of the reaction solution was record at 510 nm after 20 min of dark incubation. The peroxide value (PV) was calculated with cumene hydroperoxide as the standard.

### 2.9 *In vitro* gastrointestinal digestion

Following the method of Minekus et al. (31), approximately 4 g of the CCAs-emulsion gel were placed in a triangular flask and shaken at 3,250 rpm for 12 s to simulate oral chewing. Next, 20 mL gastric digestive juices (containing 4,000 U/mL of pepsin and 2 mg/mL of sodium chloride, pH 1.5 ± 0.1) were poured. The mixture was stirred at 720 rpm and 37 °C. After 1 h, the digestion suspension was mixed with 20 mL of simulated intestinal fluid (composed of 300 U/mL lipase, 800 U/mL of trypsin, 9.8 mg/mL of bile sodium, and 10 mol/L CaCl_2_), and adjusted to pH 7.0 ± 0.1. The digestion reaction was continued at 720 rpm and 37 °C for 2 h. Next, the digestive juices (1 mL) were aspirated and centrifuged at 4,350 *g* for 0.5 h. The free amino acid content in the supernatant was measured using the *o*-phthaldialdehyde method (32). To prepare *o*-phthaldialdehyde working solution, 1 mL of *o*-phthaldialdehyde (dissolved in methanol, 40 mg/mL) was mixed with 25 mL of tetraboronic acid (10 mmol/L), 2.5 mL of sodium dodecyl sulfonate (20 mg/mL), and 100 μL of β-mercaptoethanol. The mixture was made up to 50 mL with deionized water. Subsequently, the *o*-phthaldialdehyde working solution (4 mL) was mixed with the sample solution (200 μL), incubated at 37 °C for 2 min, and then determined at 340 nm. The standard curve was plotted using *L*-leucine (0.2–10 mmol/L).

### 2.10 Preparation of the emulsion gels loading curcumin

Based on the modified method of Xing et al. (4), curcumin was dissolved in refined corn oil (5 mg/mL), and then mixed with CCA solution (4.5 g/100 mL) at a volume ratio of 1:3 (v/v) and 3 mL of sodium alginate (2 g/100 mL). The mixture was homogenized at 20,000 rpm for 120 s with HTX-II rapid dispersion device. Immediately, calcium chloride powder was added to reach content of 5.54 mg/mL. The suspension was homogenized at 20,000 rpm for 120 s, heated at 80 °C for 30 min, and then left at 4 °C for 10 h to form an emulsion gel loading curcumin.

### 2.11 Functionality and stability of the emulsion gel loading curcumin

#### 2.11.1 Encapsulation efficiency and loading capacity of curcumin in emulsion gel

Approximately 1 g of CCA-emulsion gels loading curcumin was cut into pieces and immersed into 5 mL of anhydrous ethanol (4). The suspension was vortexed at 3,000 r/min and 30 °C to break the emulsion. After 3 min, the mixture was then centrifuged at 8,000 *g*, and the supernatant was determined at 470 nm. A standard curve with curcumin was drawn, and the encapsulation efficiency, and curcumin loading capacity of curcumin were calculated using the Equations 5 and 6:

$$\text{Encapsulation effciency}\;(\%)=W_{2}/W_{1}\times 100\%$$ (5)

$$\text{Loading capacity}\;(\%)=W_{3}/W_{2}\times 100\%$$ (6)

where, W_1_, W_2_, and W_3_ are the curcumin weights in the sample, refined corn oil, and the curcumin-loaded emulsion gel, respectively.

#### 2.11.2 Photostability of curcumin

The emulsion gel loading curcumin was exposed to UV light for 16 h, and then mixed with 3 mL of methanol (4). The mixture was vortexed at 3,000 r/min and 25 °C for 3 min, and then centrifuged at 10,000 *g*. The absorbance of the supernatant was measured at 470 nm, and the content of curcumin was calculated according to the standard curve. The curcumin in the refined corn oil was used as the control.

#### 2.11.3 Bioaccessibility of curcumin in the emulsion gels

Following the method of Xing et al. (4), the curcumin-loading emulsion gels (approximately 4 g) were placed in a triangular flask and shaken at 3,250 rpm for 12 s to simulate oral chewing. The *in vitro* digestion conducted according to the process as shown in section 2.9.7. The curcumin-loading emulsion gel pieces were digested by 40 mL gastric digestive juices (containing 4,000 U/mL of pepsin and 2 mg/mL of sodium chloride) at pH 1.5 ± 0.1 and 37 °C for 1.5 h, and then digested by 40 mL of the simulated intestinal fluid (composed of 300 U/mL lipase, 800 U/mL of trypsin, 9.8 mg/mL of bile sodium, and 10 mol/L CaCl_2_) at pH 7.0 ± 0.1 and 37 °C for 2.5 h. Here, 0.1 mol/L of NaOH was used to maintain the pH value at 7.0 ± 0.1. Subsequently, the digestive fluid was centrifuged at 4,350 *g* for 20 min, and the content of curcumin in the supernatant was measured. The bioaccessibility of curcumin was calculated using the Equation 7:

$$\text{Bioaccessiblity of curcumin}\;(\%)=W_{2}/W_{1}\times 100\%$$ (7)

where, *W_2_* and *W_1_* are the curcumin in the emulsion gel and the supernatant, respectively.

### 2.12 Statistical analysis

The repeat time for every test was three or more. Statistical analysis was conducted on the Version 16 IBM SPSS Statistics software (Chicago, IL, United States), the data significance and *post-hoc* multiple comparison was performed with one-way analysis of variance. At least three repeats/sets were used for statistical analysis. Significant level set at *p* < 0.05.

## 3 Results and discussion

### 3.1 The solubility and interface sorption ability of CCAs

**Figure 1:**
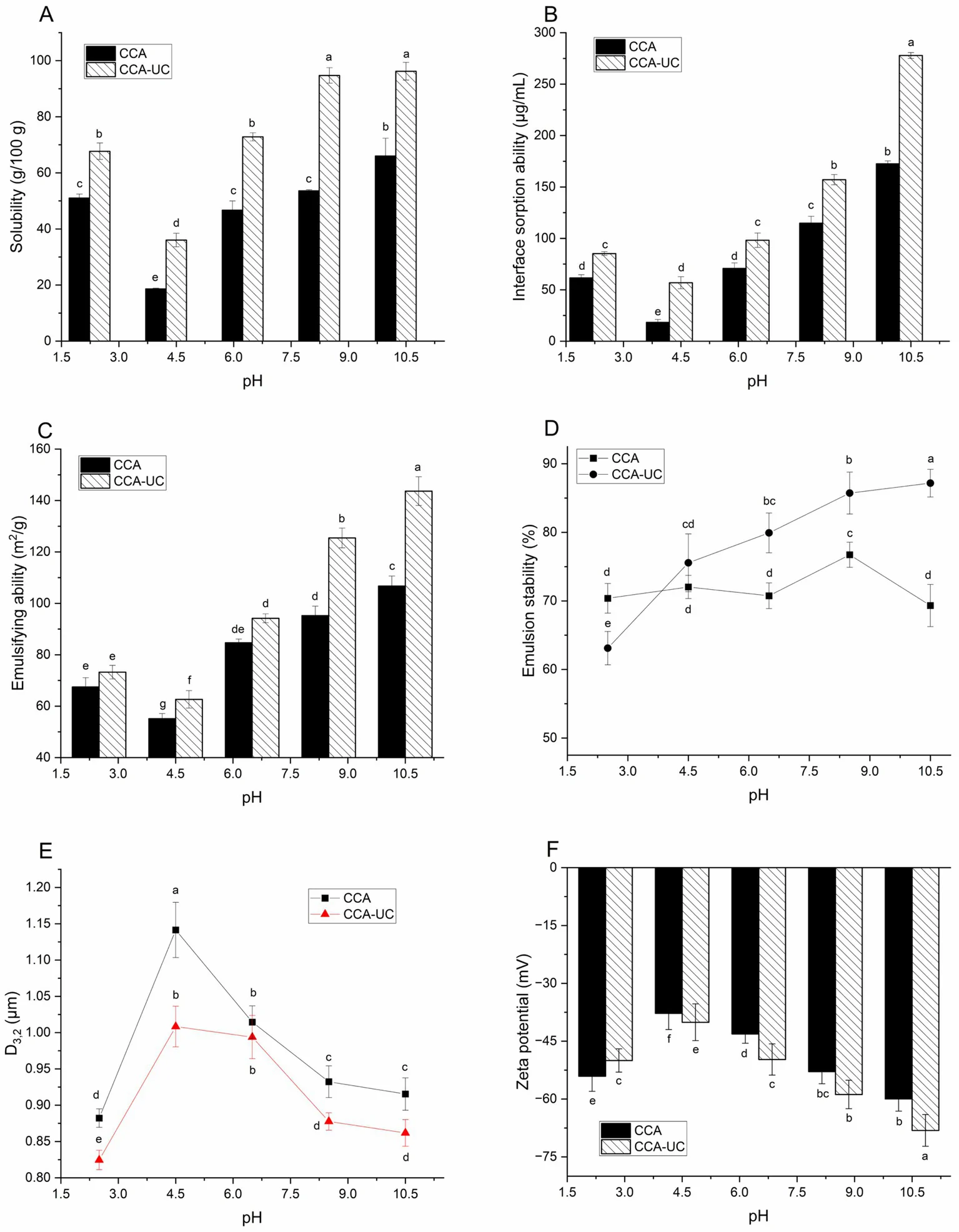
The solubility **(A)**, interface sorption capacity **(B)**, emulsifying capacity **(C)** and emulsion stability **(D)** of CCA and CCA-UC; and the particle size **(E)** and zeta-potential **(F)**, of the emulsion based on CCA and CCA-UC. CCA, coconut cake albumin; CCA-UC, coconut cake albumin modified by ultrasonic and carboxymethylation. Different lowercase letters (a–g) on the bars or data points indicate statistically significant difference (*p* < 0.05).

The degree of substitution of CCA-UC was 8.84% ± 0.47%, indicating that carboxymethyl groups have been introduced in CCA. As shown in Figure 1A, ultrasonic and carboxymethylation significantly improved the solubility of CCA (*p* < 0.05). Ultrasonic treatment caused breakdown of the amino bonds within polypeptides and exposed more hydrophilic groups (33); and the –COOH in the introduced carboxymethyl groups was hydrophilic (20), both enhanced the solubility of CCA. Moreover, CCA and CCA-UC exhibited increasing solubility when pH value increased from 4.5 to 10.5; and they showed the lowest solubility at pH 4.5, which was near the isoelectric point of coconut albumin (15). At the isoelectric point, the net charge of CCA was nearly zero and the repulsive forces between protein molecules was low (22), resulting in poor solubility. When pH increased, the charged groups gradually dissociated and the net charge increased, the binding ability of CCAs to water enhanced, and the hydration membrane thickened, resulting in superior solubility (34). Moreover, ultrasonic and carboxymethylation increased the interface sorption ability of CCA (*p* < 0.05) (Figure 1B). Ultrasonic and carboxymethylation exposed more hydrophilic groups from CCA (Figure 1A) and increased the steric hindrance, expanding the structure, and enhancing its adsorbing ability on the water–oil interface (30). Furthermore, the –CH_2_–COOH in carboxymethyl group increased the affinity of CCA to oils (14), thereby improving its interface sorption ability.

### 3.2 Effects of the modifications on the emulsifying properties

As depicted in Figures 1C,D, the emulsion stability and emulsifying capacity of CCA were lower than those of CCA-UC (*p* < 0.05). Ultrasonication and carboxymethylation increased the solubility of CCA, enhanced its interface sorption ability, thereby improving its affinity to water and oil, reducing the surface tension, resulting in a superior emulsifying capacity. Moreover, increased interface sorption capacity promoted the formation of a stable film surrounding the droplets (25), thereby making the emulsion more stable. Furthermore, the emulsifying capacity of CCA increased as pH raised from 4.5 to 10.5, mainly ascribed to the increased solubility and interface sorption ability (Figures 1A,B); while the increased zeta-potential and reduced particle size of the emulsion droplets (Figures 1E,F) enhanced the emulsion stability of CCA and CCA-UC as pH raised from 4.5 to 8.5.

### 3.3 Zeta-potential and particle size of the CCA-emulsions

Emulsions with lower particle size exhibit better stability (25). As shown in Figures 1E,F, the CCA-UC-emulsion showed smaller particle size and higher zeta-potential than CCA-emulsion gel at pH 2.5–10.5 (*p* < 0.05). Ultrasonication and carboxymethylation enhanced the interface sorption ability and emulsifying capacity of CCA (Figures 1B,C), increasing its ability to form emulsion film surrounding the oil droplets, which can hinder aggregation of the droplets (4), thereby increasing the zeta-potential and reducing the emulsion particle size. Moreover, the higher solubility of CCA-UC (Figure 1A) indicated that more protein can take part in stabilizing the emulsion, the dissociate of protein was enhanced and the net charge increased (35), thereby increasing repulsive force between the droplets, resulting in higher zeta-potential and smaller particle size. Additionally, alkali pH-shift made the structure of proteins more extended and promoted dissociation (22), which was conducive to increasing the net charge and repulsive forces between the droplets, leading to higher zeta-potential and smaller size. Coincidentally, CCA- and CCA-UC-emulsion exhibited the largest size and lowest zeta-potential at pH 4.5 (near its isoelectric point) (19), corresponding to their lowest solubility and emulsifying properties (Figures 1A,C,D).

### 3.4 Structural characteristics of the emulsion gels

#### 3.4.1 Morphology

**Figure 2:**
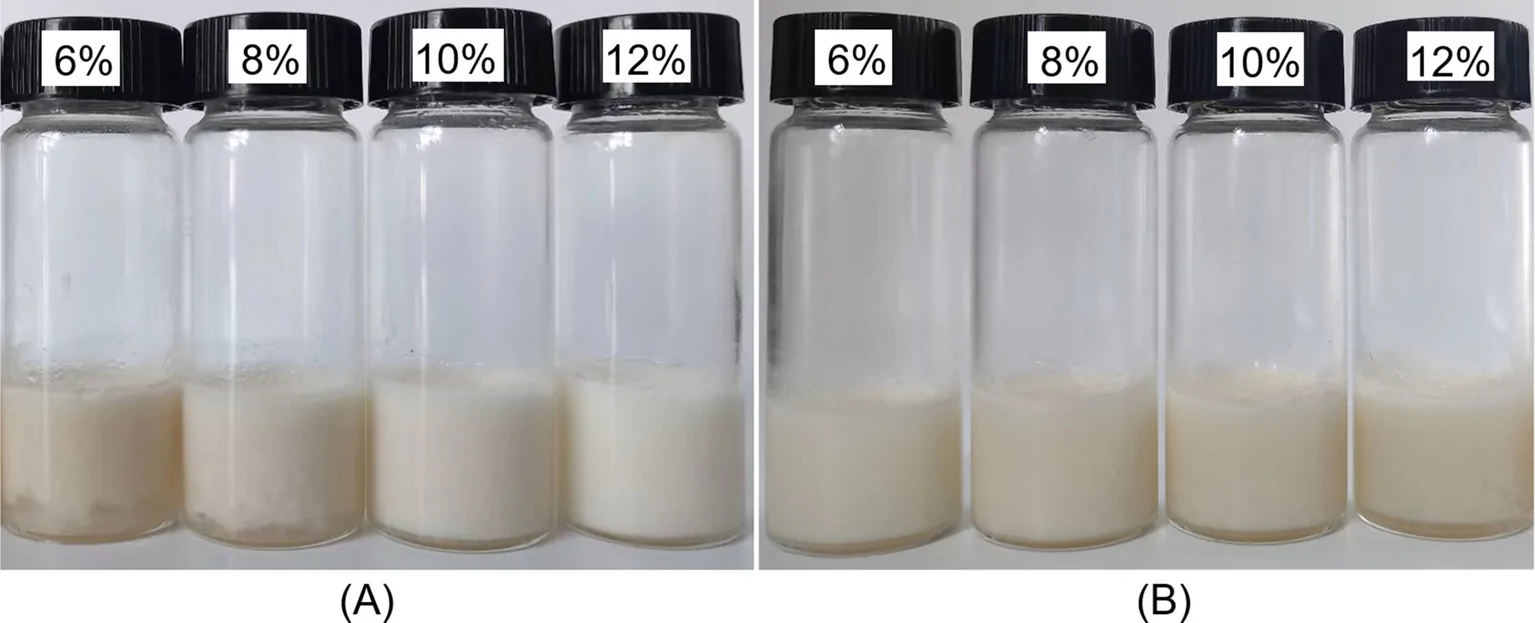
The morphologies of the emulsion gels based on CCA **(A)** and CCA-UC **(B)** after storage for 12 h at addition amount of 6–12 g/100 g.

As shown in Figure 2A, at dose of 10–12 g/100 g, CCA formed fine and stable emulsion gels; however, when the amount was 6–8 g/100 g, layering phenomenon occurred on the side within the gels after storage for 12 h. At lower dose, the thickness of the films formed by CCA surrounding the oil droplets was not enough to stable the lipid phase, and the interaction forces (hydrophobic effects and hydrogen bonds) within the gels were relatively weak, resulting in an unstable emulsion gel structure (11). By contrast, CCA-UC formed more stable emulsion gel at dose of 6–12 g/100 g (Figure 2B), for ultrasonication and carboxymethylation enhanced the water–oil interface sorption capacity and emulsion stability of CCA (Figures 1B,D).

#### 3.4.2 Electron microscope

**Figure 3:**
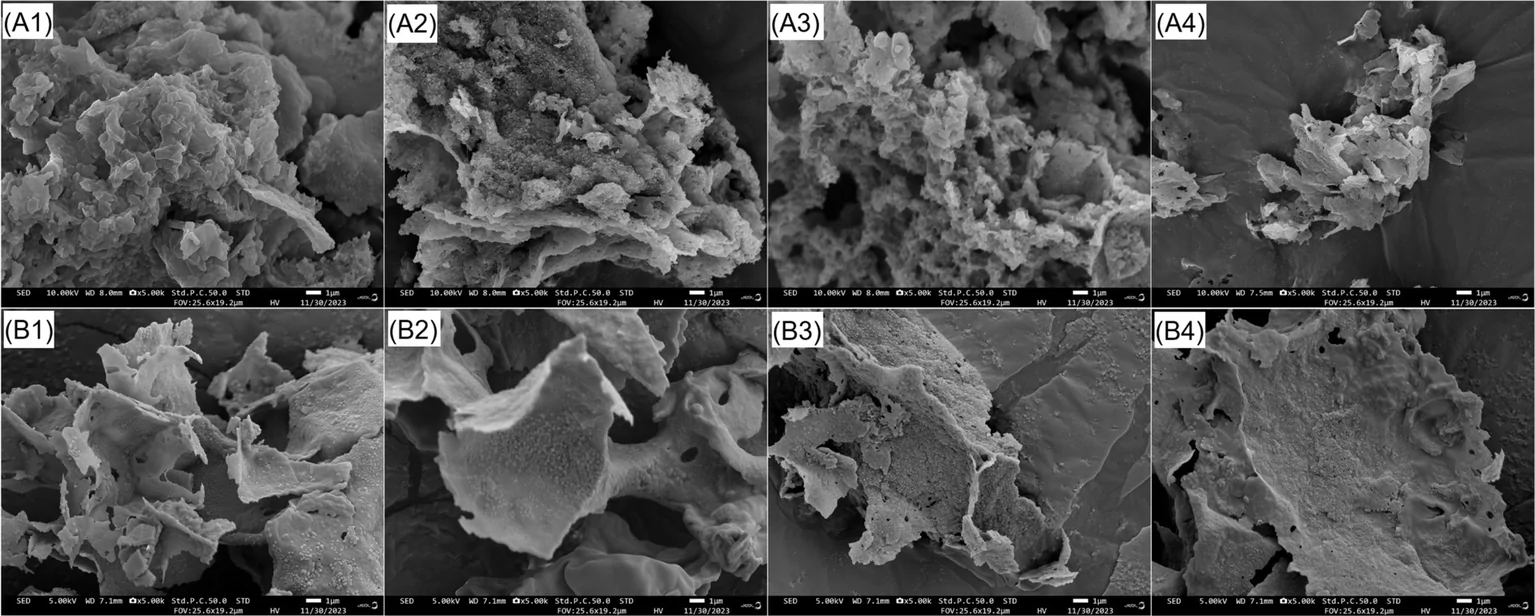
The scanning electron micrographs of the emulsion gels based on CCA **(A1–4)** and CCA-UC **(B1–4)** at addition amount of 6–12 g/100 g. The magnification for scanning electron micrographs was 5,000 × with a scale bar of 1 μm.

The surface structure of CCA-emulsion gels was rough and presented a multi-layered stacking (Figures 3A1–4); by contrast, CCA-UC-emulsion gels displayed a more compact, smooth, and orderly structure (Figures 3B1–4). The reason for this phenomenon was that ultrasonic and carboxymethylation improved the solubility, interface sorption capacity, and emulsifying properties (Figures 1A–D), the thickness, toughness, and elasticity of the film surrounding the droplets were increased consequently (22), which was helpful for forming stable and delicate gel structure during gelation. The –COOH and –CH_3_ in carboxymethyl groups enhanced the affinity of CCA to water and oils, respectively, leading to a more compact gel network structure. Moreover, the introduced carboxymethyl groups increased the steric hindrance between CCA molecules, increasing the structural flexibility, and facilitating the further formation of a cross-linked network structure during the cooling process (30). Additionally, the microstructure of CCA-UC-emulsion gels became smoother as dose increased, for increase in amount increased the thickness of the film surrounding oil droplets, and enhanced the interaction forces (Table 1), resulting in a more compact gel structure. Similar results were obtained by Kamer (27).

**Table 1:** Intermolecular interactions of the emulsion gels based on coconut cake albumin (CCA) and coconut cake albumin modified by ultrasonication and carboxymethylation (CCA-UC) with different amount.

Addition amount (g/100 g)	Disulfide bonds	Hydrogen bonds	Hydrophobic interactions	Ionic bonds
Emulsion gel based on CCA				
6	0.09 ± 0.01a	0.25 ± 0.04d	0.45 ± 0.05f	0.12 ± 0.02c
8	0.11 ± 0.01a	0.34 ± 0.04c	0.59 ± 0.06e	0.17 ± 0.01b
10	0.12 ± 0.02a	0.42 ± 0.04bc	0.67 ± 0.09d	0.20 ± 0.03b
12	0.09 ± 0.01a	0.47 ± 0.05b	0.79 ± 0.04c	0.21 ± 0.04b
Emulsion gel based on CCA-UC				
6	0.11 ± 0.01a	0.39 ± 0.04c	0.51 ± 0.06ef	0.19 ± 0.01b
8	0.08 ± 0.01a	0.49 ± 0.06b	0.74 ± 0.06c	0.24 ± 0.00b
10	0.09 ± 0.01a	0.57 ± 0.04a	1.02 ± 0.07b	0.27 ± 0.02a
12	0.07 ± 0.01a	0.62 ± 0.06a	1.25 ± 0.12a	0.29 ± 0.01a

Different little letters (a–d) in the same column represent significant differences (*p* < 0.05).

#### 3.4.3 FT-IR spectroscopy

**Figure 4:**
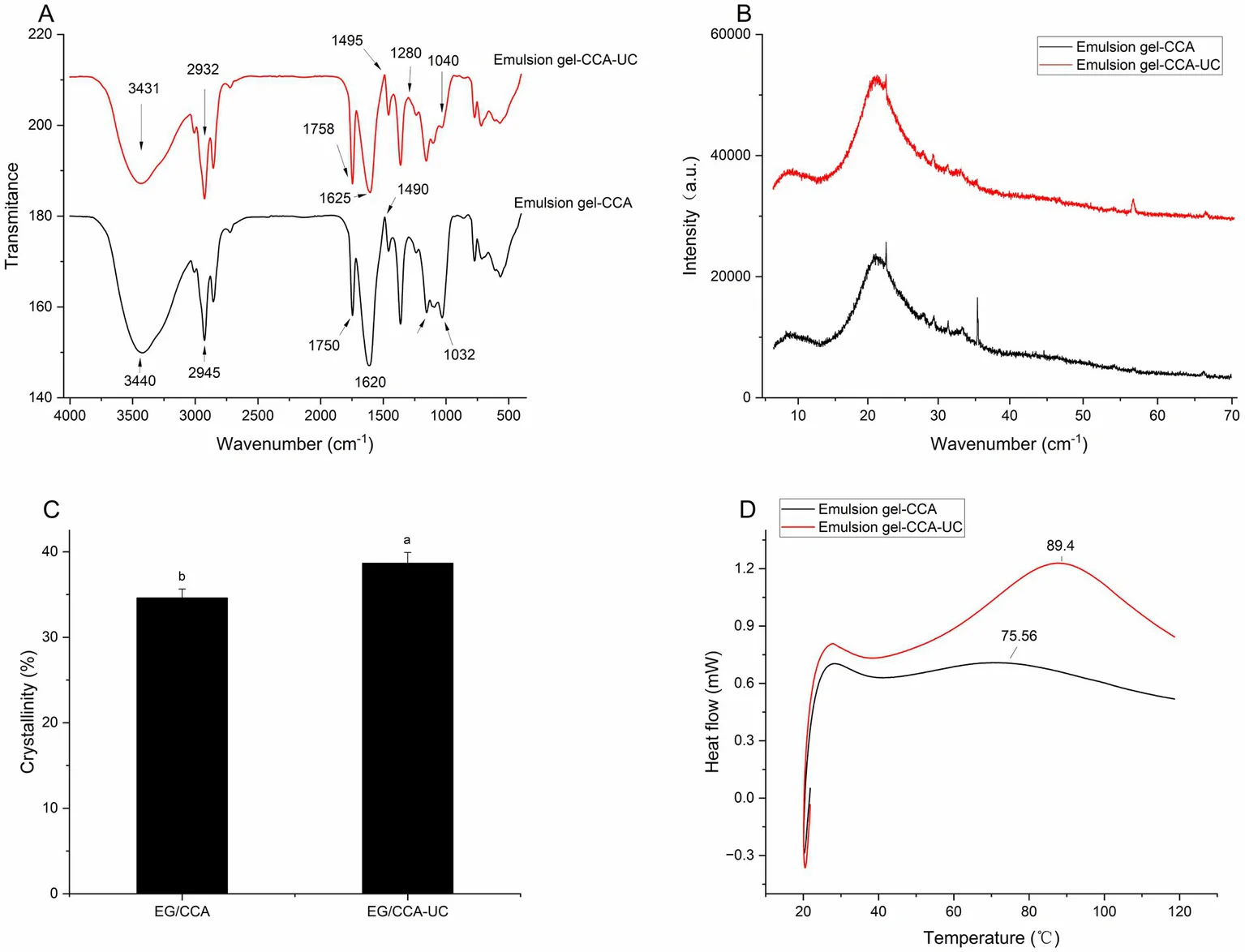
The structural characteristics of the emulsion gels based on CCA and CCA-UC. **(A)** Fourier-transform infrared spectroscopy **(A)**; *X-*ray diffraction patterns **(B)**; crystallinity **(C)**; and differential scanning calorimeter spectroscopy **(D)**. Different small letters (a, b) above on bars mean significant difference (*p* < 0.05).

In the FT-IR spectra of CCA- and CCA-UC-emulsion gels (Figure 4A), the absorption peaks at approximately 3,440, 1750, and 1,032 cm^−1^ corresponding to shaking of C–N, carboxyl group, and N–H, respectively (10, 36), reflecting that amide bond, carboxyl and amino groups were broken, and CCA and CCA-UC underwent denaturation during ultrasonic and heating process, the linkages of the polypeptide chains were broken, and the structure became extended, consequently, a gel structure was formed during the further cooling process (4). More important, ultrasonication and carboxymethylation increased the hydrophilic regions of the gel, promoting the formation of an amphiphilicity gel. Furthermore, in the spectrum of CCA-UC emulsion gel, red or blue shift observed on the peaks at 1758, 2932, and 1,280 cm^−1^ were due to the vibrations of carboxyl group, carbonyl group, and –CH_3_, respectively (12, 28), indicating the introduction of carboxymethyl groups in MPC-UC. Similar results were obtained by Zhang et al. (13).

#### 3.4.4 XRD analysis of emulsion gels

As shown in Figure 4B, the emulsion gels based on CCA and CCA-UC showed two distinct crystallization peaks at 2*θ* = 9° and 20°, respectively, indicating that in the amorphous region they have a semi-crystalline structure (35). The emulsion gel based on CCA-UC showed a superior crystallinity compared to CCA-emulsion gel (*p* < 0.05) (Figure 4C), due to improvement of hydrophilicity after ultrasonication and carboxymethylation. Hydrogen bond is one key force that form and maintain the crystal structure of emulsion gels (12). Enhancing hydrophilicity increased the affinity of CCA-UC to water or sodium alginate, which was helpful to forming more intermolecular hydrogen bonds, leading to a higher crystallinity. Abou-Elsoud et al. (33) found that increase in hydrophobicity enhanced the crystallinity of lipoprotein-emulsion gel.

#### 3.4.5 Intermolecular forces distribution

As shown in Table 1, hydrophobic forces and hydrogen bonds were the main intermolecular interactions within CCA- and CCA-UC-emulsion gels. Since coconut cake albumin lacked sulfhydryl amino acids (Met and Cys) (19), disulfide bond was very low. Alternatively, the content of hydrophilic amino acids, especially Arg, Glu, and Asp was high (17), which was conducive to form intramolecular and intermolecular hydrogen bonds during gelation process (2). Compared with CCA-emulsion gel, CCA-UC-emulsion gels exhibited higher content of hydrophobic interactions, hydrogen bonds, and ionic bonds at concentration of 8–12 g/100 g. The structural flexibility of CCA was increased after ultrasonication and carboxymethylation (22), more functional groups such as carboxyl, carbonyl, hydroxyl, and other hydrophobic or hydrophilic groups were exposed or introduced, which was helpful to forming hydrogen bonds and hydrophobic interactions between polypeptides during gel formation (1). Moreover, the introduced carboxymethyl groups improved the hydrophilicity of CCA (Figure 1A), thereby increasing the ionic bonds within CCA-UC-emulsion gel. Furthermore, the content of hydrogen and ionic bonds was one reason for the higher crystallinity of CCA-UC-emulsion gel (Figure 4C).

#### 3.4.6 DSC analysis

Figure 4D shows the DSC profiles of CCA- and CCA-UC-emulsion gels. Obviously, CCA-UC-emulsion gel displayed a higher transition temperature (89.4 °C) than CCA-emulsion gel, suggesting that ultrasonication and carboxymethylation increased the thermal stability of CCA-emulsion gel. Ultrasonication and carboxymethylation increased the ability of CCA to form more stable and compact gel structure (Figure 2) and increased the crystallinity of the CCA stabilized emulsion gel (Figure 4C), leading to superior thermal stability. Moreover, the intramolecular forces, especially higher hydrogen bonds and hydrophobic interactions (Table 1) enhanced the resistance of CCA-UC-emulsion gel to decomposition at elevated temperature. Ultrasonication and carboxymethylation improved the thermal stability of lipoprotein gel and millet fibres, respectively (14, 33).

### 3.5 Characterization of the emulsion gels

#### 3.5.1 Water-holding capacity

**Figure 5:**
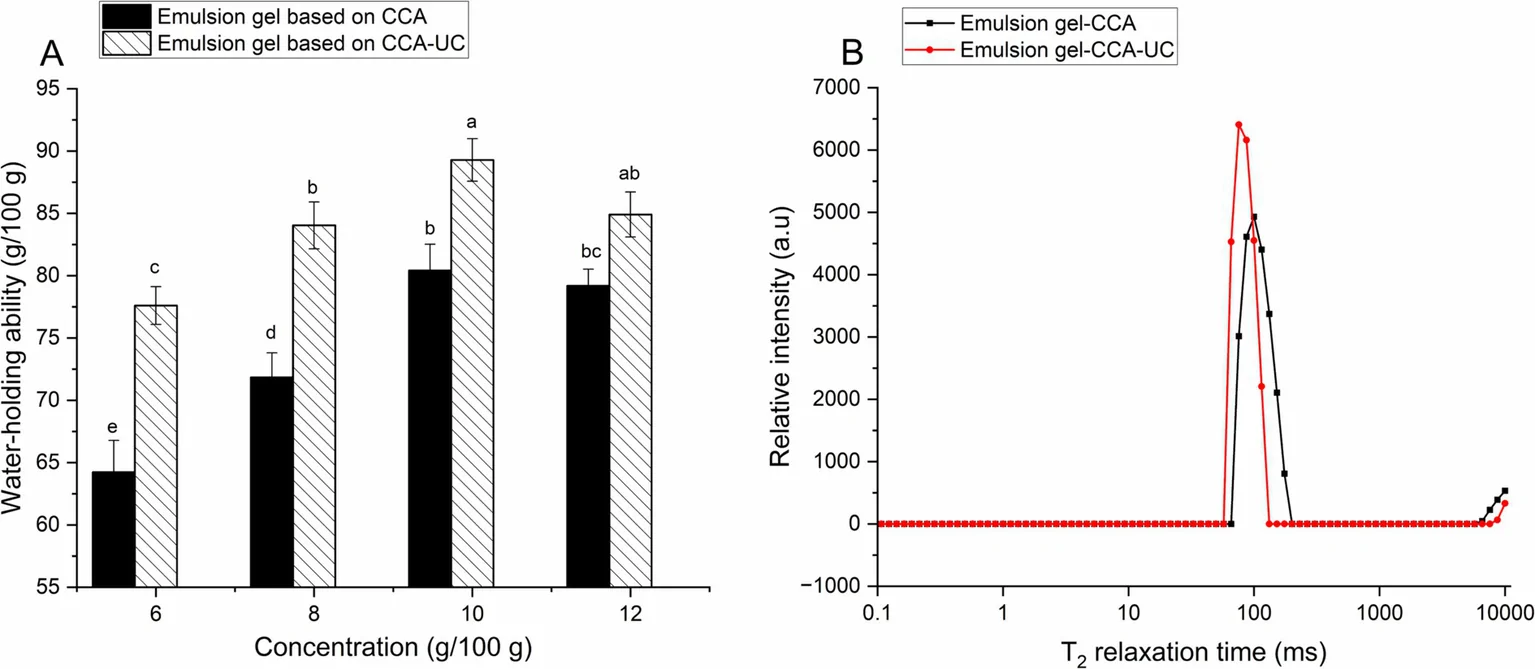
The water holding capacity **(A)** and water distribution **(B)** of the emulsion gels based on CCA and CCA-UC. Different lowercase letters (a–e) on the bars indicate statistically significant difference (*p* < 0.05).

Compared with CCA-emulsion gel, CCA-UC-emulsion gel exhibited higher WHC when the concentration was 6–12 g/100 g (*p* < 0.05) (Figure 5A). Improvement of hydrophilicity can increase the affinity of polypeptides to water (2). Ultrasonication and carboxymethylation increased the hydrophilicity of CCA, thereby enhancing the WHC of CCA emulsion gel. Moreover, ultrasonic and carboxymethylation made the microstructure of the gel more compact and denser (Figures 3B1–4), thereby increasing the WHC (4). Furthermore, the WHC of CCA- and CCA-UC-emulsion gels increased as the concentration increased from 6 to 10 g/100 g. Increased concentration increased the hydrogen and ionic bonds within CCA-emulsion gel, enhancing its affinity to water. Coincidentally, Hegde et al. (37) found that ultrasound improved the WHC of millet protein-emulsion gel.

#### 3.5.2 Water distribution in the emulsion gels

In the Low-Field Nuclear Magnetic Resonance spectra of the emulsion gels based on 10 g/100 g of CCA and CCA-UC, the T_22_ (1–100 ms) and T_23_ (>100 ms) indicated the bound and free water within gels, respectively (12). The CCA-emulsion gel mainly contained free water and a low content of bound water (Figure 5B); while the content of bound water increased within CCA-UC-emulsion gel, perhaps due to their superior WHC (Figure 4A) and high content of hydrogen bonds (Table 1). Ultrasonication and carboxymethylation improved the hydrogen bonds, crystallinity, and emulsifying capacity of CCA (Table 1 and Figures 1C, 4C), and enhanced its ability to form compact and stable network gel structure (Figures 2, 3), thereby increasing the affinity of the emulsion gel to solidify water (30). Moreover, carboxymethylation increased the hydrophilicity of CCA (Figure 1A), further enhancing the affinity of CCA-UC-emulsion gel to water, leading to a higher content of bound water. Han et al. (12) found that carboxymethylation improved the WHC of bean protein-emulsion gel.

#### 3.5.3 Textural properties

As shown in Table 2, the springiness, gumminess, and hardness of CCA- and CCA-UC-emulsion gels increased as the dose increased from 6 to12 g/100 g. An increase in dose increased the abound water content and enhanced the intermolecular forces (Figure 5B and Table 1), resulting in a higher hardness and cohesiveness. Since gumminess is the product of hardness and cohesiveness; and chewiness is the product of gumminess and springiness (2), an increase in hardness and cohesiveness resulted in superior gumminess and chewiness. Furthermore, the hydrophobic effect and hydrogen bonds were enhanced as the dose increased (Table 1), leading to aggregation of proteins and stronger interactions between the gel matrixes, contributed to the higher gumminess, cohesiveness, and springiness (13).

**Table 2:** The textural properties of the emulsion gels based on different concentration of CCA and CCA-UC.

Concentration (g/100 g)	Hardness (g)	Springiness	Cohesiveness	Gumminess	Chewiness (g)	Resilience
Emulsion gels based on CCA						
6	32.97 ± 1.35e	0.64 ± 0.06e	0.51 ± 0.01d	16.08 ± 0.53e	17.46 ± 1.39d	0.18 ± 0.01a
8	35.32 ± 0.74e	0.67 ± 0.03e	0.53 ± 0.07d	19.32 ± 1.34de	16.87 ± 2.00d	0.16 ± 0.02a
10	47.71 ± 3.65d	0.75 ± 0.06d	0.69 ± 0.05c	24.38 ± 1.25 cd	27.64 ± 1.64b	0.16 ± 0.00a
12	56.81 ± 0.22c	0.80 ± 0.05c	0.74 ± 0.02bc	25.42 ± 2.56 cd	29.07 ± 0.13b	0.16 ± 0.02a
Emulsion gels based on CCA-UC						
6	41.02 ± 1.91de	0.74 ± 0.08d	0.64 ± 0.04c	22.45 ± 1.72d	18.54 ± 1.17 cd	0.15 ± 0.00a
8	46.93 ± 0.78d	0.79 ± 0.03c	0.77 ± 0.00b	29.99 ± 2.56c	21.28 ± 1.38c	0.16 ± 0.02a
10	61.90 ± 3.49b	0.85 ± 0.07b	0.89 ± 0.08a	31.45 ± 0.97bc	33.16 ± 0.44ab	0.18 ± 0.01a
12	67.44 ± 2.42a	0.90 ± 0.02a	0.85 ± 0.06a	36.37 ± 1.64a	35.83 ± 2.74a	0.19 ± 0.02a

Different little letters (a–e) represent significant differences (*p* < 0.05).

CCA-UC-emulsion gels exhibited superior hardness, springiness, gumminess, cohesiveness, and chewiness than CCA-emulsion gel (*p* < 0.05). Springiness indicates the ability of emulsion gels to return to their original state after the withdrawal of strain (11); and cohesiveness reflects the intermolecular binding forces within the gels and determines integrity during swallowing (2). Ultrasonication and carboxymethylation enhanced the emulsifying capacity of CCA (Figure 1C) and increased the hydrophobic effect and hydrogen bons within the gel (Table 1), improving its ability to form a stable gel structure, thereby increasing the cohesiveness and gumminess (11), further improving its springiness, resilience, and chewiness. Moreover, the higher content of bound water (Figure 5B), more stable and denser microstructure (Figure 3B) attributed to the higher springiness, gumminess, and resilience of CCA-UC-emulsion gels. Additionally, CCA-UC-emulsion gel exhibited higher hardness (67.44) and chewiness (35.83 g) than those of the egg white protein-emulsion gel (4), indicating its potential as textural improver.

#### 3.5.4 Rheological characteristics

**Figure 6:**
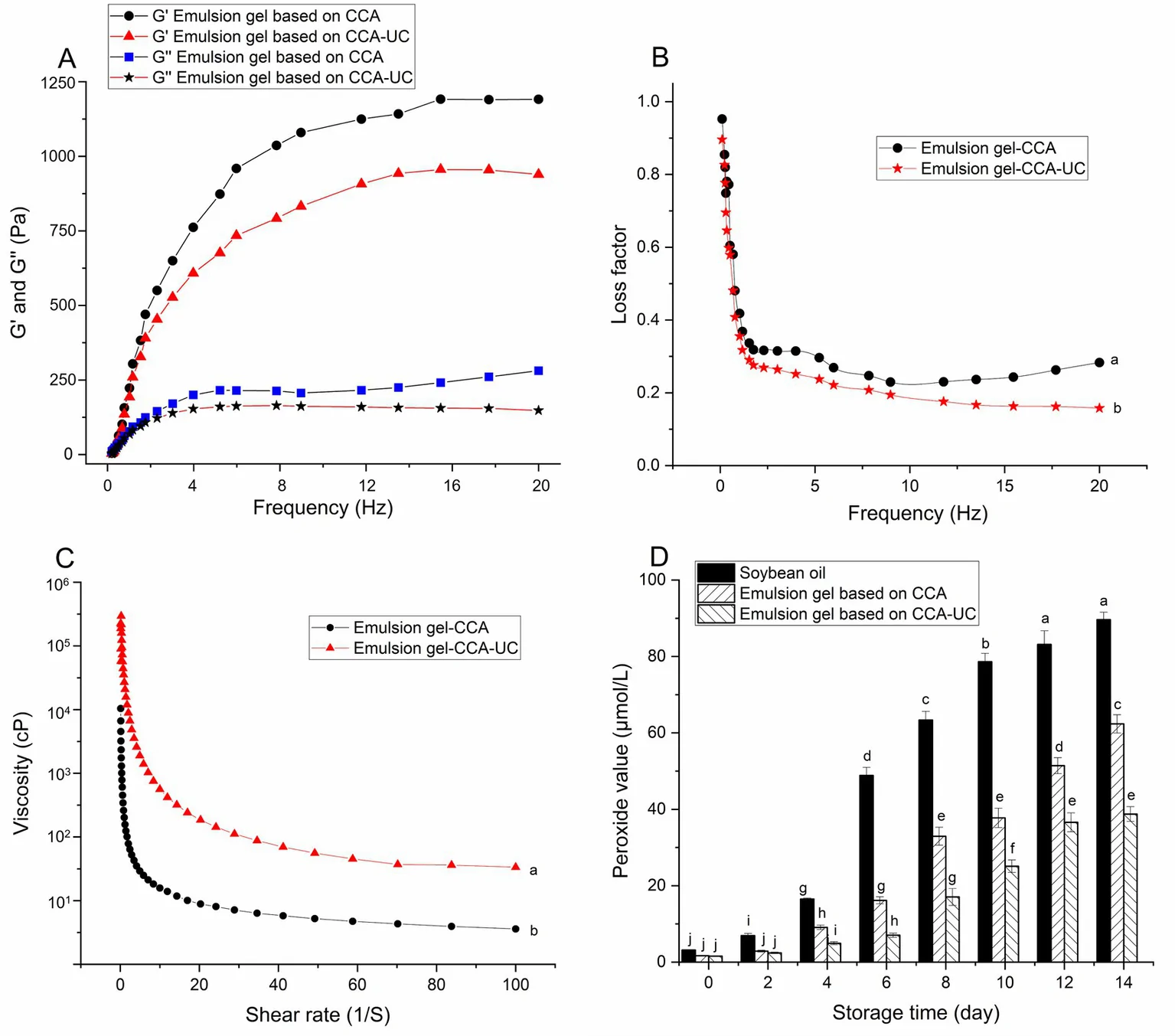
The rheological properties and oxidant stability of the emulsion gels based on CCA and CCA-UC. **(A)** The energy storage modulus (*G’*) and loss modulus (*G”*), and **(B)** loss factor at various frequency; **(C)** viscosity at different shear rate; and **(D)** peroxide values during storage time. Different lowercase letters (a-j) on the bars mean significantly difference (*P* < 0.05).

As frequency increased, the *G’* and *G”* of CCA- and CCA-UC-emulsion gels increased (Figure 6A) and their loss factor (tanδ = *G”*/*G’*) decreased (Figure 6B). The *G”* was lower than *G’* and the loss factor value was always less than 1, suggesting that these emulsion gels had semisolid-like network structure which can resist reversible and irreversible deformation (29). Moreover, the viscosity of these emulsion gels lowered with increasing shear rate (Figure 6C), but this trench was not sharp, evidencing that these emulsion gels have a stable network structure, endowing them adjustable elasticity and good stability (36). The higher viscosity contributed to the higher cohesiveness of CCA-UC-emulsion gel (Table 2).

Moreover, compared to CCA-emulsion gel, CCA-UC-emulsion gel exhibited lower *G*’, *G*” and loss factor, and higher viscosity, showing that ultrasonic and carboxymethylation enhanced the elasticity and stability of the gel (25). Ultrasonication and carboxymethylation were helpful for CCA to form stable emulsion film surrounding the droplets, and further establish denser emulsion gel structure during the cooling process. The higher crystallinity (Figure 4C), superior hardness, and resilience, and cohesiveness of CCA-UC-emulsion gel (Table 1) evidenced this. Furthermore, CCA-UC-emulsion gel exhibited higher bound water content and WHC (Figure 5), contributing to their higher viscosity and elasticity, and lower loss factor (29). The rheological properties of millet gliadin emulsion were improved by carboxymethylation (22).

### 3.6 Lipid oxidation of the emulsion gels

The POV values of CCA- and CCA-UC-emulsion gels were lower than those of soybean oil (control) from the second storage day (*p* < 0.05) (Figure 6D), indicating that these emulsion gels effectively hindered the oxidation of lipid. First, the physical protection effect of the gels’ compact and stable network structure scattered the light and reduced the touching rate between oxygen and the lipid. Second, some amino acid residues, such as Arg, Phe, and Tyr in coconut albumin can quench free radicals and inhibit the oxidation of lipid (19). Furthermore, CCA-UC-emulsion gel exhibited lower POV value than CCA-emulsion gel from the fourth day (*p* < 0.05), suggesting that ultrasonic and carboxymethylation significantly enhanced the oxidative stability of CCA-emulsion gel. Wang et al. (22) found that carboxymethylation enhanced the antioxidant activity of millet gliadin emulsion. However, more special mechanism needs further investigation.

### 3.7 *In vitro* digestion of the emulsion gels

**Figure 7:**
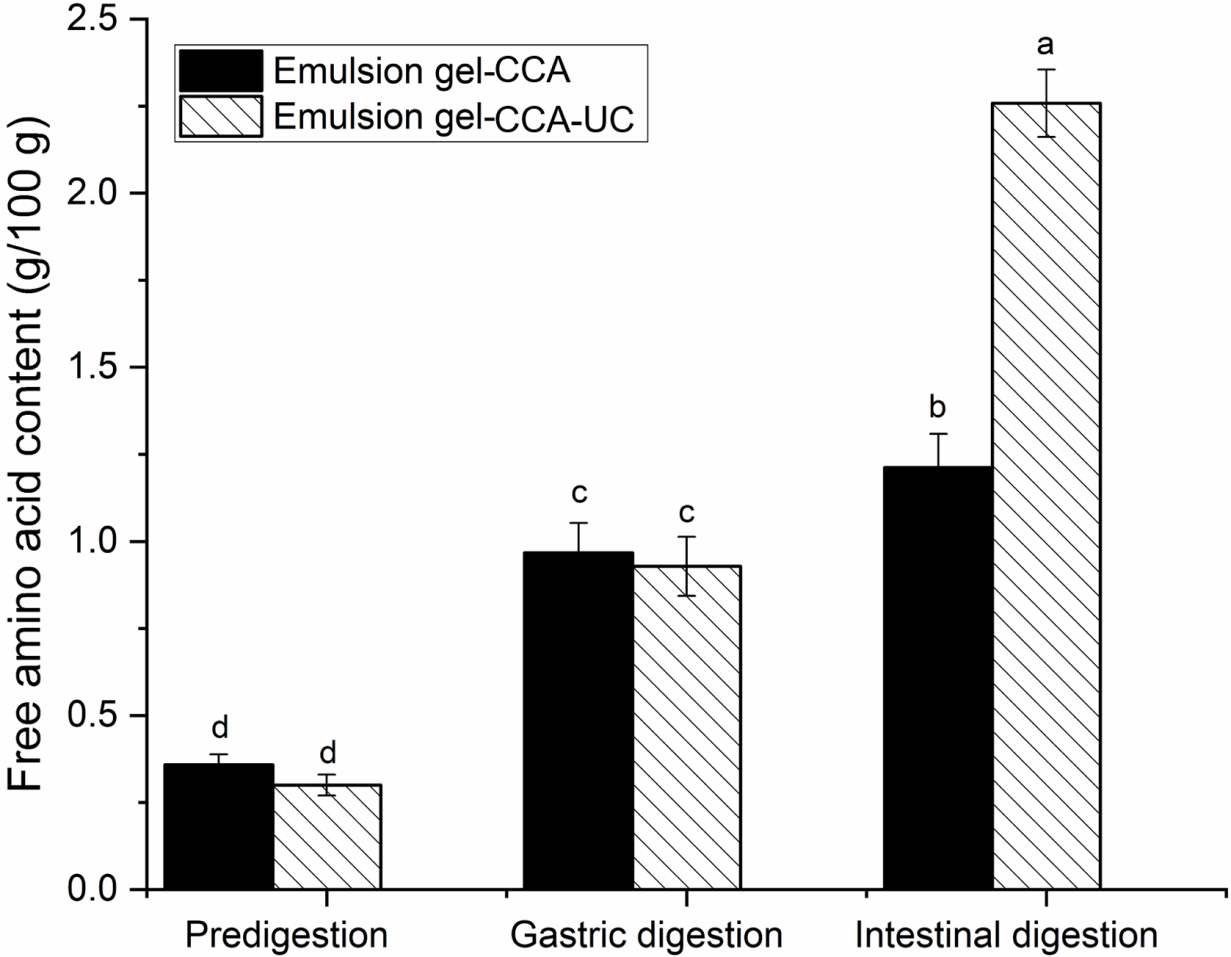
The gastrointestinal digestion of the emulsion gels based on CCA and CCA-UC. Different lowercase letters (a–d) on the bars or data points indicate statistically significant difference (*p* < 0.05).

Both CCA- and CCA-UC-emulsion gels exhibited a lower digestion rate in the stomach than in the intestine (*p* < 0.05) (Figure 7), for there was lower amount of protease in stomach. Alternatively, the emulsion gels are hydrolyzed by pancreatic lipase, and the amino bonds are hydrolyzed by trypsin, chymotrypsin, aminopeptidase, and dipeptidase in the intestine (37). CCA-UC-emulsion gel exhibited a lower gastric digestion rate but a higher intestinal digestion rate compared with CCA-emulsion gel, (Figure 7) (*p* < 0.05), indicating that ultrasonic and carboxymethylation enhanced the gastric digestion resistance of CCA-emulsion gel and the intestinal release rate. The more compact and denser gel structure (Figure 2), stronger intermolecular forces (Table 1), and superior hardness (Table 2) of CCA-UC-emulsion gels were responsible to their lower gastric digestion. Moreover, ultrasonic and carboxymethylation increased the emulsifying properties of CCA (Figures 1C,D), promoting the amino acids produced from the hydrolysis of CCAs to surround the oil droplets and hinder oil aggregation (29), increasing the contacting rate between oil and lipase, leading to a higher digestion.

### 3.8 Photostability of curcumin in the emulsion gels

**Figure 8:**
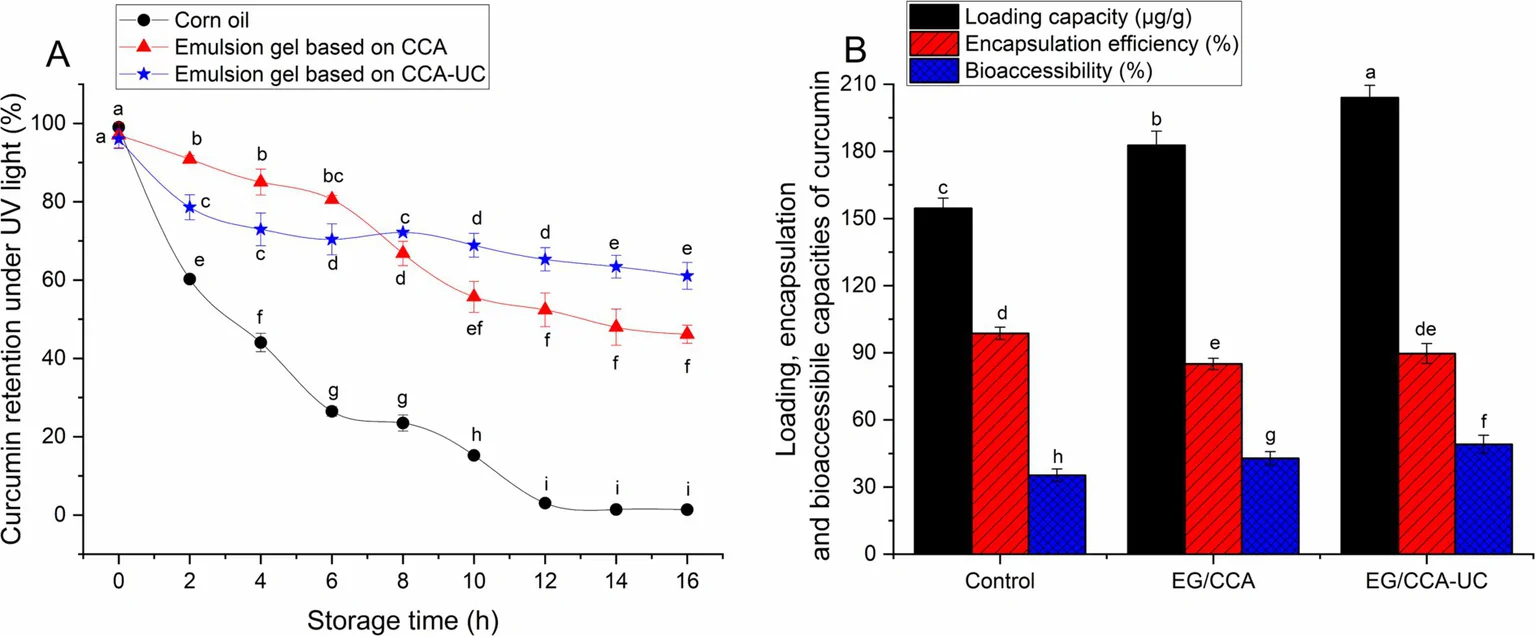
The encapsulation capacity, loading ability, and bioaccessibility **(A)** and photostability **(B)** of curcumin in coil oil and the emulsion gels based on CCA and CCA-UC. Different lowercase letters (a–i) on the bars or data points denote statistically significant difference (*p* < 0.05).

**Figure 9:**
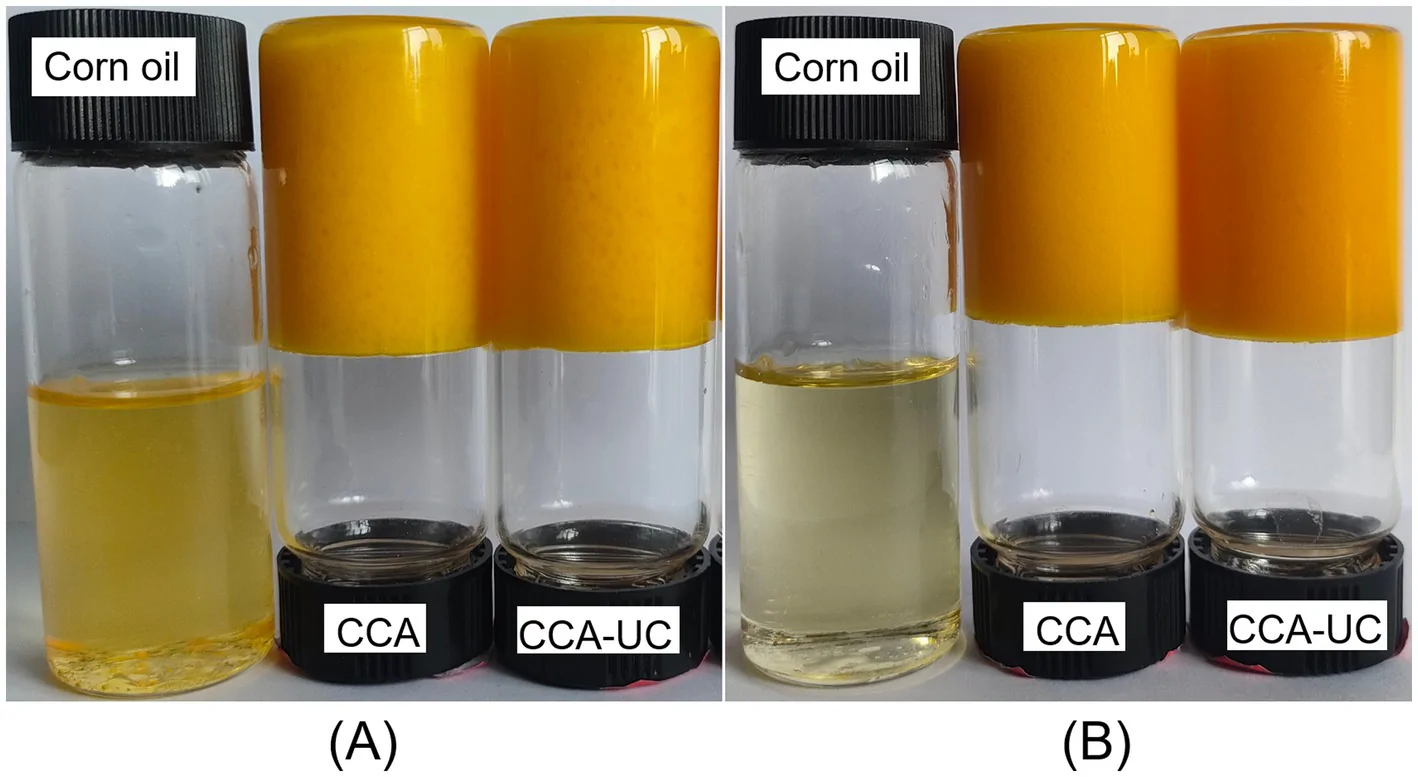
The morphology of curcumin in corn oil, CCA- and CCA-UC-emulsion gels before **(A)** and after 16 h of ultraviolet ray treatment **(B)**.

Curcumin is unstable under ultraviolet rays, which is one reason for its poor bioaccessibility (39). Figure 8A depicts the curcumin photostability in CCA- and CCA-UC-emulsion gels was better than in corn oil (*p* < 0.05). Figures 9A,B depict the morphology of curcumin in corn oil, CCA-and CCA-UC-emulsion gels before and after 16 h of ultraviolet ray treatment. It was obvious that after 16 h of ultraviolet ray treatment the curcumin in corn oil degraded and its color faded away; whereas the curcumin in CCA-and CCA-UC-emulsion gels were more stable (Figure 9B). The curcumin in corn oil confronted ultraviolet rays directly, easily leading to decomposition and a lower retention (40). There were three main reasons for the better photostability of curcumin in CCA-emulsion gels: (I) the oil droplets containing curcumin were surrounded by dense CCA and CCA-UC, which can effectively shade the ultraviolet rays (41), thereby reducing the decomposition of curcumin; (II) CCA- and CCA-UC-emulsion gels exhibited considerable antioxidant activity (Figure 6D), effectively inhibiting the oxidation of curcumin caused by ultraviolet rays; and (III) the emulsion gels based on CCAs with high hardness, cohesiveness, and WHC (Table 2 and Figure 5), providing a physical barrier which can scatter ultraviolet rays. Water in emulsion gels can scatter lights (38). Begum et al. (17) found that the emulsion gels based on alginate improved the encapsulation capacity of curcumin. Moreover, the curcumin photostability in CCA-UC-emulsion gel was better than in CCA-UC-emulsion gel during the 10th to 16th days (*p* < 0.05), ascribed to the denser compact structure (Figure 2), stronger intermolecular forces (Table 1), higher bound water content (Figure 5), and superior antioxidant activity of CCA-UC-emulsion gel (Figure 6D).

### 3.9 Encapsulation efficiency and loading capacity of curcumin

As depict in Figure 8B, CCA- and CCA-UC-emulsion gels exhibited excellent encapsulation efficiency (84.97 and 89.64%) and loading efficiencies (182.68 and 204.01 μg/g), which were equal to those of the emulsion gels based on egg white protein and soybean protein (4, 42) and their curcumin-loading capacity were higher than the control (corn oil), indicating their potential as curcumin delivery system. Compared with corn oil, the stable gel network structure of CCA- and CCA-UC-emulsion gels, their high hydrophobic effects and physical protective effects were suitable to encapsulate curcumin. Moreover, CCA-UC-emulsion gel exhibited superior encapsulation and loading efficiencies than CCA-emulsion gel (*p* < 0.05). After ultrasonication and carboxymethylation, the content of hydrophobic effect and disulfide bonds increased (Table 1), which was helpful to enhance the affinity of the gels to curcumin (5). Additionally, compared with CCA, CCA-UC formed a more compact and stable emulsion gel network structure with higher cohesiveness (Figure 2 and Table 2), contributed to the higher encapsulation and loading rate of curcumin (42). These effects collectively increased the curcumin loading and encapsulation activities of CCA-emulsion gel.

### 3.10 Bioaccessibility of curcumin

CCA- and CCA-UC-emulsion gels exhibited superior bioaccessibility of curcumin than the control (coil oil) (*p* < 0.05) (Figure 8B), corresponding the results of Xing et al. (4) and Guo et al. (1). Gastric stability and intestinal release rate of curcumin were crucial for its bioaccessibility (27). The stability of curcumin in soybean oil can be decreased by pH-shifting, food compositions, and other factors in the intestine (4), resulting in a poor bioaccessibility. By contrast, during gastrointestinal digestion of CCA, free amino acids and peptides were produced consequently, which surrounded the oil droplets and prevent oil aggregation (37), promoting the contacting between oil and lipases and decomposition of the oils, resulting in higher release rate and bioaccessibility of curcumin. Furthermore, curcumin in CCA-UC-emulsion gel displayed higher bioaccessibility than that in CCA-emulsion gel (*p* < 0.05), mainly ascribed to its better gastric stability and intestinal digestion (Figure 7). Since ultrasonication and carboxymethylation enhanced the intestinal digestion of CCA-emulsion gel, decreasing the gastric hydrolysis of curcumin and increasing intestinal releasing rate, thereby promoting the adsorption of curcumin in intestinal. Farooq et al. (8) found that the emulsion gels based on camellia oil and gum Arabic improved the bioaccessibility of curcumin.

## 4 Conclusion

The results of this study demonstrated that ultrasonication and carboxymethylation improved the emulsifying properties of CCA by enhancing its solubility and interface sorption capacity, increasing zeta-potential, and reducing droplets size. Moreover, ultrasonication and carboxymethylation enhanced hydrophobic effects and hydrogen bonds within CCA-emulsion gel, and enhanced its crystallinity, bound water content and viscosity, reduced the loss factor, thereby making its structure more stable, resulting in superior oxidant stability, elastically, cohesiveness, hardness, and chewiness. Furthermore, ultrasonication carboxymethylation inhibited the gastric digestion but promoted the intestinal digestion of CCA-emulsion gel, and improved the curcumin photostability, encapsulation and loading capacities, and bioaccessibility. However, more specific mechanisms should be investigated in the further work.

## Data Availability

The original contributions presented in the study are included in the article/Supplementary material, further inquiries can be directed to the corresponding author.
